# Pseudomyogenic Hemangioendothelioma of the Femur Treated by Intercalary Resection and Massive Allograft Reconstruction: A Case Report

**DOI:** 10.1155/cro/2427724

**Published:** 2026-02-10

**Authors:** Fernando N. Martín Cocilova, Elisabetta Neri, Filippo Nozzoli, Tiziana Tatti, Annarita Palomba, Giuliana Roselli, Domenico A. Campanacci

**Affiliations:** ^1^ Department of Orthopaedic Oncology and Reconstructive Surgery, Careggi University Hospital, University of Florence, Florence, Italy, aou-careggi.toscana.it; ^2^ Section of Pathology, Department of Health Sciences, University of Florence, Florence, Italy, unifi.it; ^3^ Department of Radiology, Careggi University Hospital, Florence, Italy, aou-careggi.toscana.it

**Keywords:** bone allograft, bone tumor, intercalary resection, plate fixation, pseudomyogenic hemangioendothelioma

## Abstract

Pseudomyogenic hemangioendothelioma (PHME) is a very rare vascular tumor that usually arises in the extremities, mainly cutaneous or subcutaneous, but also occurs in deeper locations, such as muscles and bone. Less than 200 cases have been reported so far, and primary intraosseous PHMEs are further infrequent. We present a clinical case of PMHE of the femur in a young male adult successfully treated by intercalary resection and massive allograft reconstruction with plates fixation. After more than 2 years of follow‐up, the patient is disease‐free and asymptomatic, walking with full weight‐bearing with radiographic evidence of allograft union.

## 1. Introduction

The 2013 World Health Organization (WHO) soft tissue tumor classification introduced pseudomyogenic (epithelioid sarcoma‐like) hemangioendothelioma (PMHE) as a new type of vascular tumor [1]. In 2021, a consensus paper from the Connective Tissue Oncology Society community of experts on the incidence threshold and the list of entities, defined it as an “Ultra‐Rare Soft Tissue Sarcoma Identified Based on Incidence,” meaning those entities with an incidence of approximately < 1 per 1,000,000, that renders extremely difficult to conduct well powered, prospective clinical studies [2]. This condition is characterized by biologically borderline malignant potential and high rates of local recurrence while it seldom metastasizes leading to favorable overall survival and prognosis [3]. It has a predilection for young male adults, and the most frequent location is the lower extremity [1, 3], even if it has been described in other anatomical regions as the oral cavity [4], thoracic wall [5], breast [6], maxillary sinus [7], or external genital organs [8].

To date, few articles have been published concerning PHME with bone involvement [9–12]. We report a case of bone PHME of the femoral diaphysis where, due to pathologic fracture, extensive osteolysis and consistent risk of local recurrence [3], intercalary resection with massive allograft reconstruction was chosen to achieve secure oncologic margins while ensuring functional joint preservation.

## 2. Case Presentation

A 21‐year‐old homeless male patient with a language barrier was admitted to our emergency department due to increasing pain and swelling on the left thigh for about 2 months. There was neither a complaint of lower limb weakness nor a history of trauma, and he was otherwise in good health.

**Figure 1:**
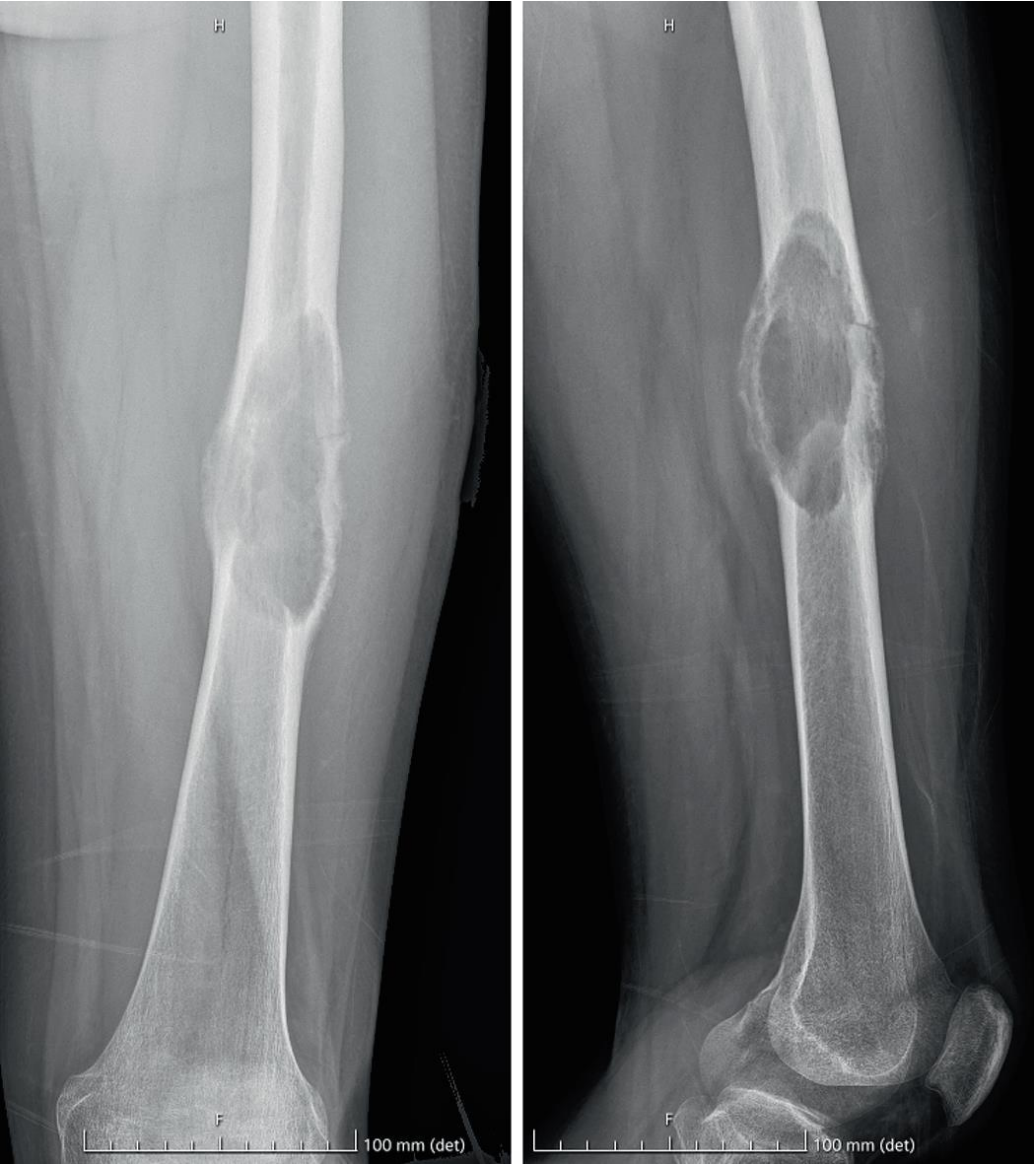
Preoperative X‐rays showed an osteolytic lesion in the middle third of the femoral shaft with aggressive features, cortical bone disruption, and undisplaced pathologic fracture.

A hard and nonmobile mass was detected in the middle third of the thigh. Blood tests showed normal procalcitonin and C‐reactive protein rates and high values of ESR. Radiographs revealed an osteolytic diaphyseal femoral lesion with aggressive features and undisplaced pathologic fracture (Figure 1).

**Figure 2:**
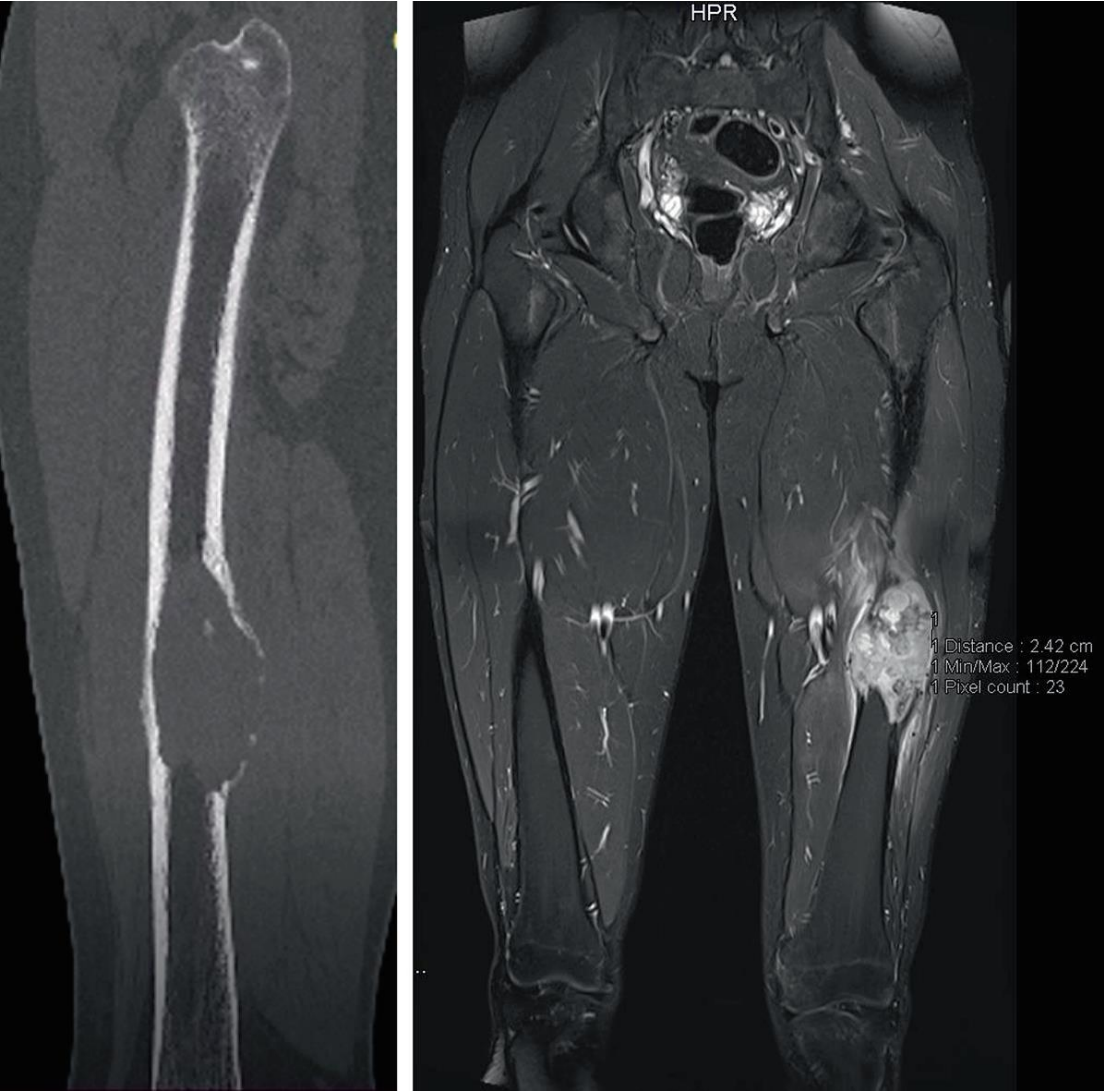
Multiplanar reformation (MPR) sagittal CT confirmed a bulge lytic bone lesion with cortical disruption (left image). Short Tau inversion recovery (STIR) MRI coronal image showed a bone‐destroying mass with heterogeneous high signal and perifocal edema of surrounding soft tissues (right image).

A direct CT scan and a contrast MRI scan were obtained to assess the extent of the lesion and its soft tissue involvement (Figure 2).

**Figure 3:**
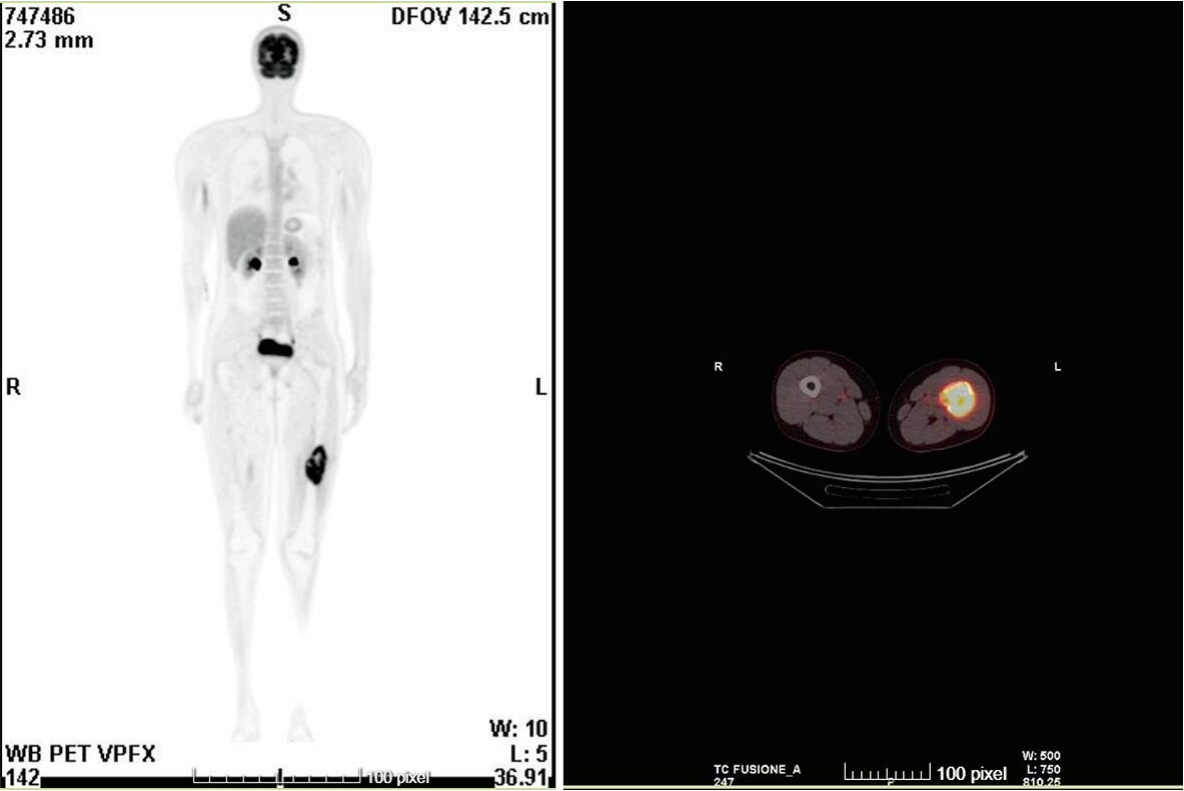
PET‐CT images detected heteroplastic tissue in the left femur and bilateral inguinal and axillary lymph nodes with phlogistic aspect.

A CT‐guided biopsy was performed. According to our pathologist, morphological and immunohistochemical (IHC) findings were compatible with the diagnosis of PHME. Staging was made by contrast‐enhanced chest–abdomen CT, revealing minor lymph nodes in retroperitoneum, groin and axillary region, as well as through a whole‐body 18F‐FDG PET‐CT scan (Figure 3).

**Figure 4:**
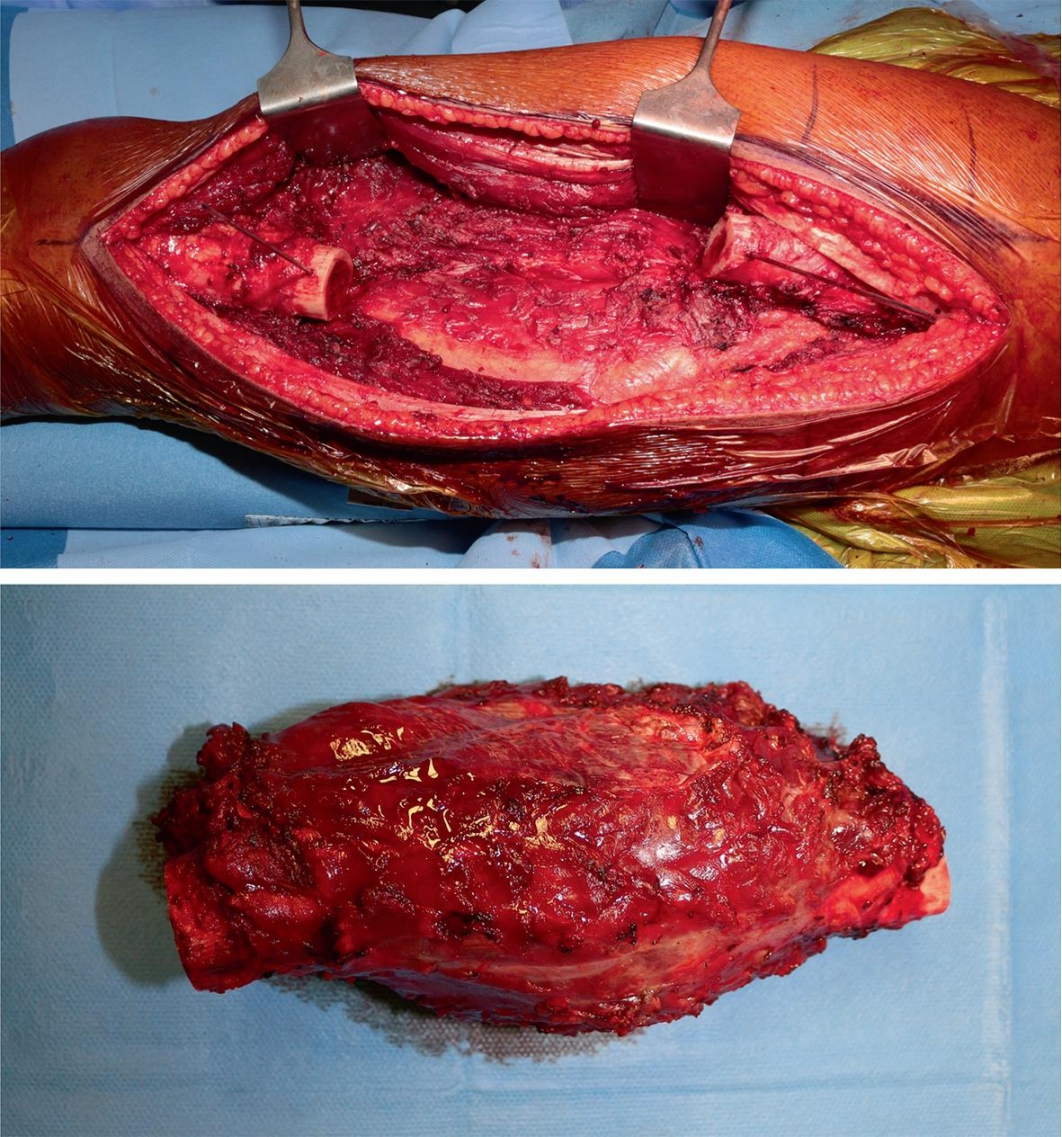
Surgical approach and tumor excision.

We discussed the case together with our multidisciplinary tumor board (MTB). Due to the tumoral bone extension with cortical disruption and pathologic fracture, we decided to treat the patient with intercalary resection and massive allograft reconstruction. An extended lateral approach was utilized. Bone resection margins were assessed with preoperative planning. Wide resection including 17 cm of bone and a portion of vastus lateralis and intermedius was performed (Figure 4).

**Figure 5:**
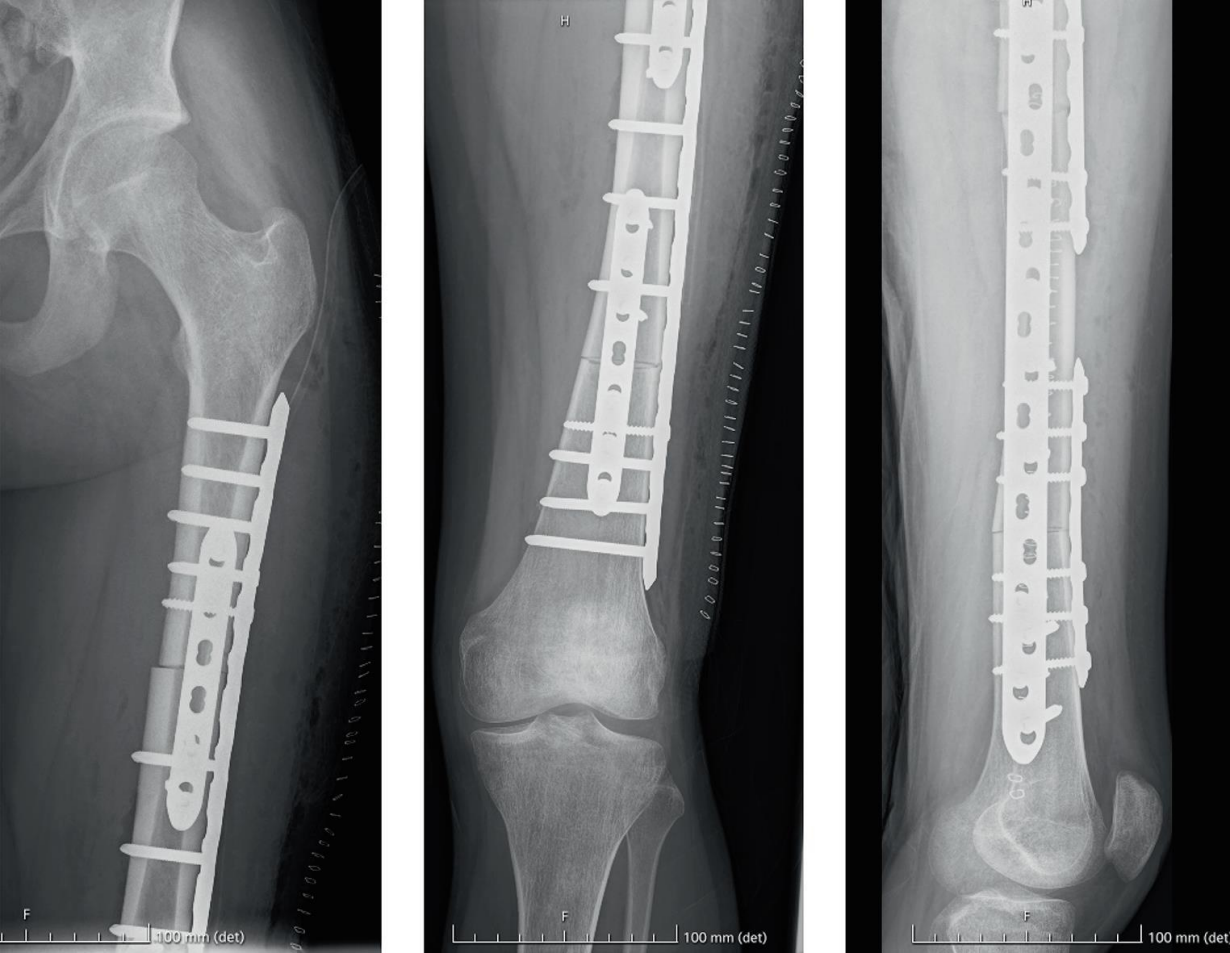
Postoperative X‐ray after intercalary allograft reconstruction.

Frozen sections of proximal and distal femoral canal revealed disease‐free margins. Then, intercalary massive allograft reconstruction was performed with a lateral plate fixation combined with two anterior plates at the proximal and distal osteotomies. Limb length restoration and optimal axial and rotational alignment were achieved (Figure 5).

The specimen was sent to Pathology Department for further histological examination. Hematoxylin and eosin (H&E) stained slides revealed a tumor composed of sheets and fascicles of plump spindle cells with abundant eosinophilic cytoplasm and eccentric nuclei, along with diffuse elements showing rhabdomyoblastic resemblance. A minor component of cells with epithelioid cytomorphology was also present. Focal reactive woven bone and scattered osteoclast‐like giant cells were observed. The tumor cells contained vesicular nuclei with generally small nucleoli. The degree of nuclear atypia was mild to moderate, mitotic activity was low, and no tumor necrosis was observed. Surgical margins were clear of tumor cells. Immunohistochemistry showed a marked expression of AE1/AE3 pan‐cytokeratin and weak and focal for the endothelial transcription factor ERG. Nuclear staining for FOSB was consistently found. Myogenin and SMA expression were negative. Morphological and IHC profiles of the tumor were consistent with the diagnosis of PHME, according to the 5th edition of the WHO Classification of Soft Tissue and Bone Tumors. H&E and immunohistochemistry findings are illustrated in Figure 6.

Figure 6Histopathological findings. H&E stained slide (a: magnification 200×, scale bar 100 *μ*m) showed a tumor composed of spindle cells arranged in loose strips and bundles, and the cells were rich and plump, with eosinophilic cytoplasm and oval nuclei, mimicking rhabdomyoblasts. Immunohistochemistry revealed FOSB nuclear positivity (b: magnification 200×, scale bar 100 *μ*m), AE1/AE3 pan‐cytokeratin (c: magnification 200×, scale bar 100 *μ*m), and endothelial transcription factor ERG (d: magnification 200×, scale bar 100 *μ*m) expression.

**Figure:**
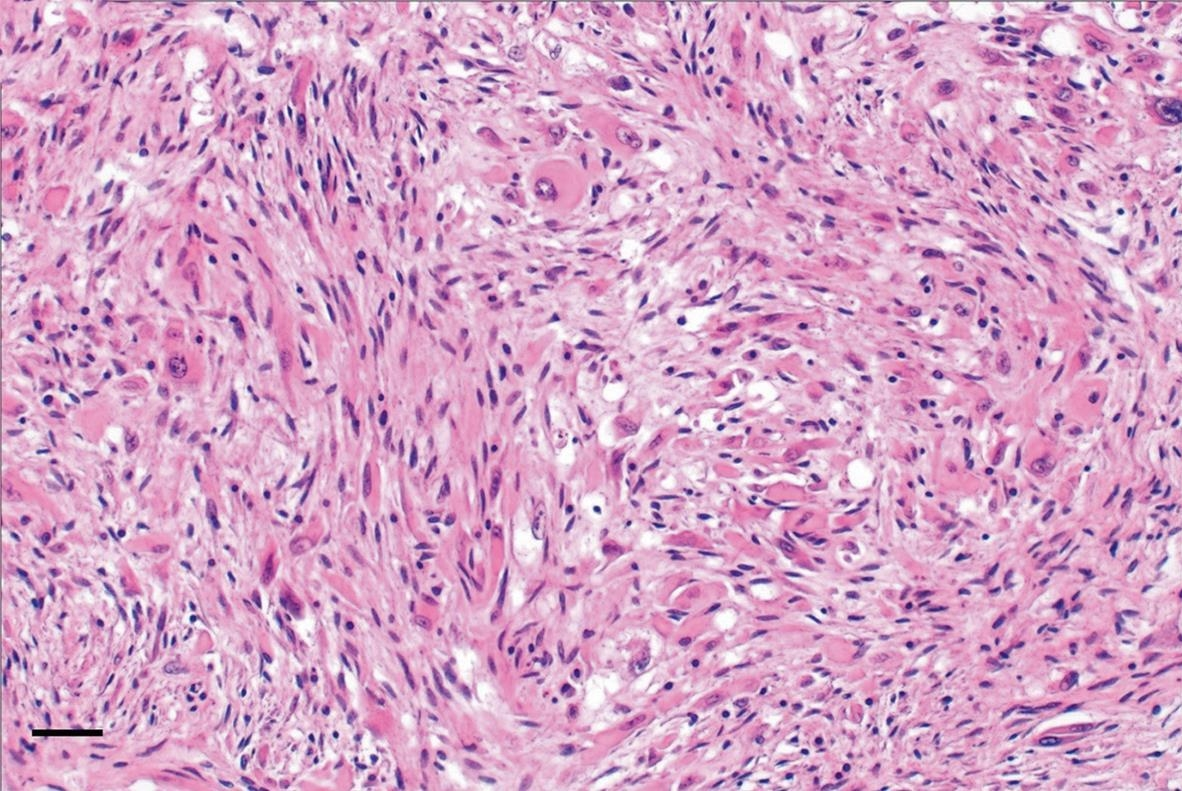
(a)

**Figure:**
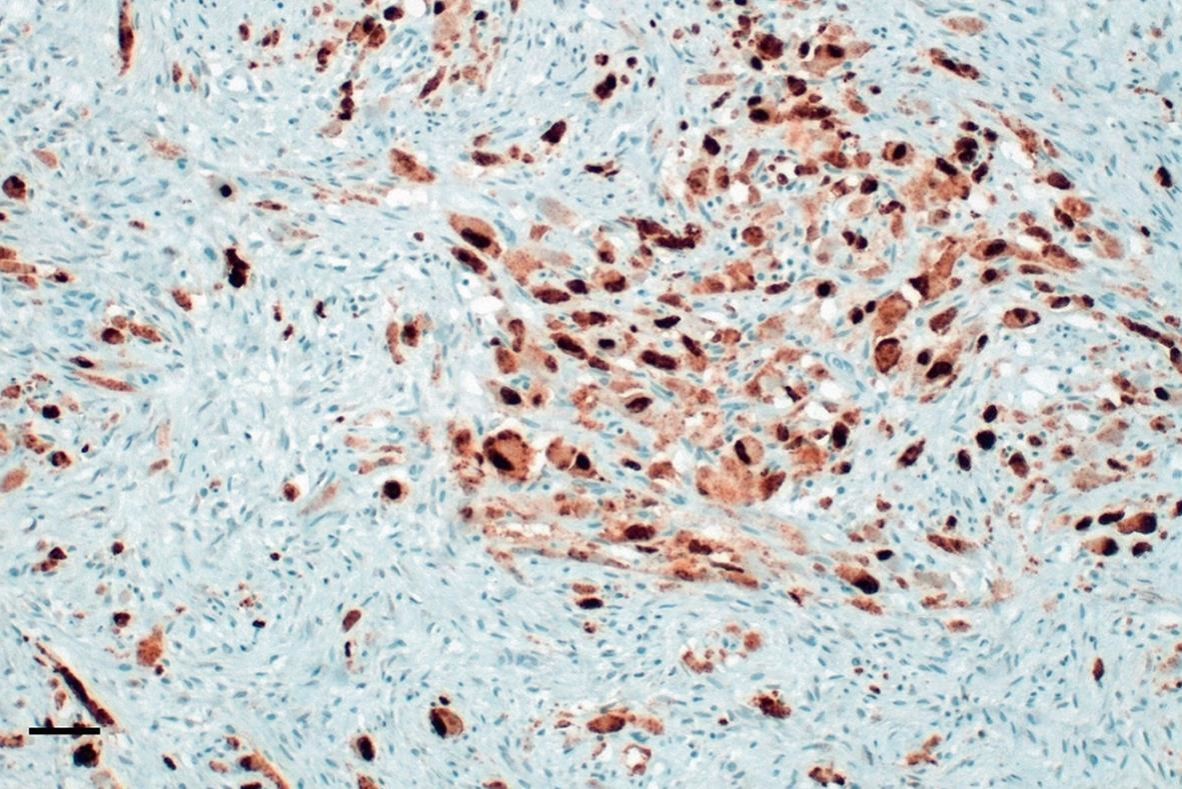
(b)

**Figure:**
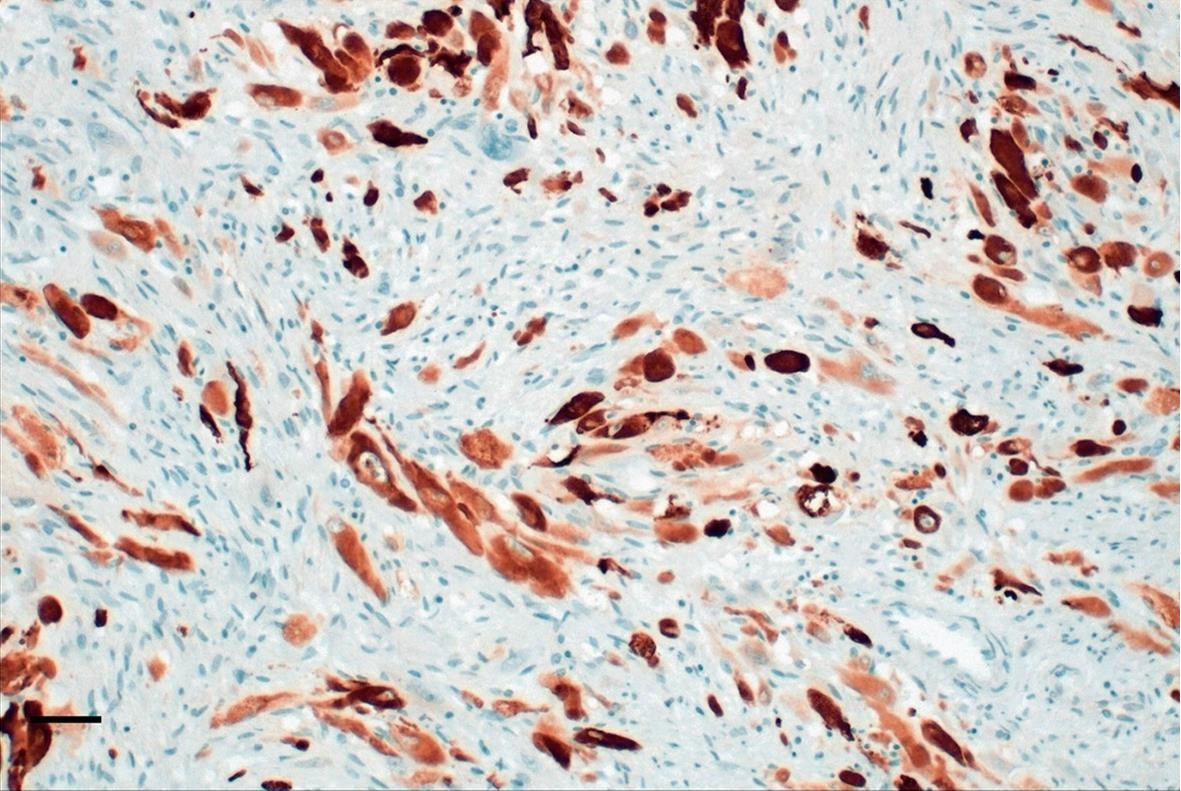
(c)

**Figure:**
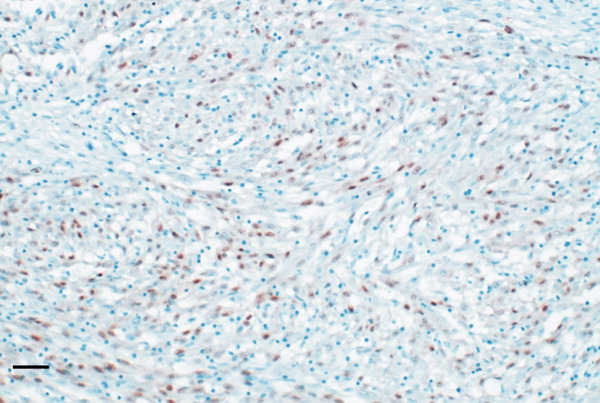
(d)

**Figure 7:**
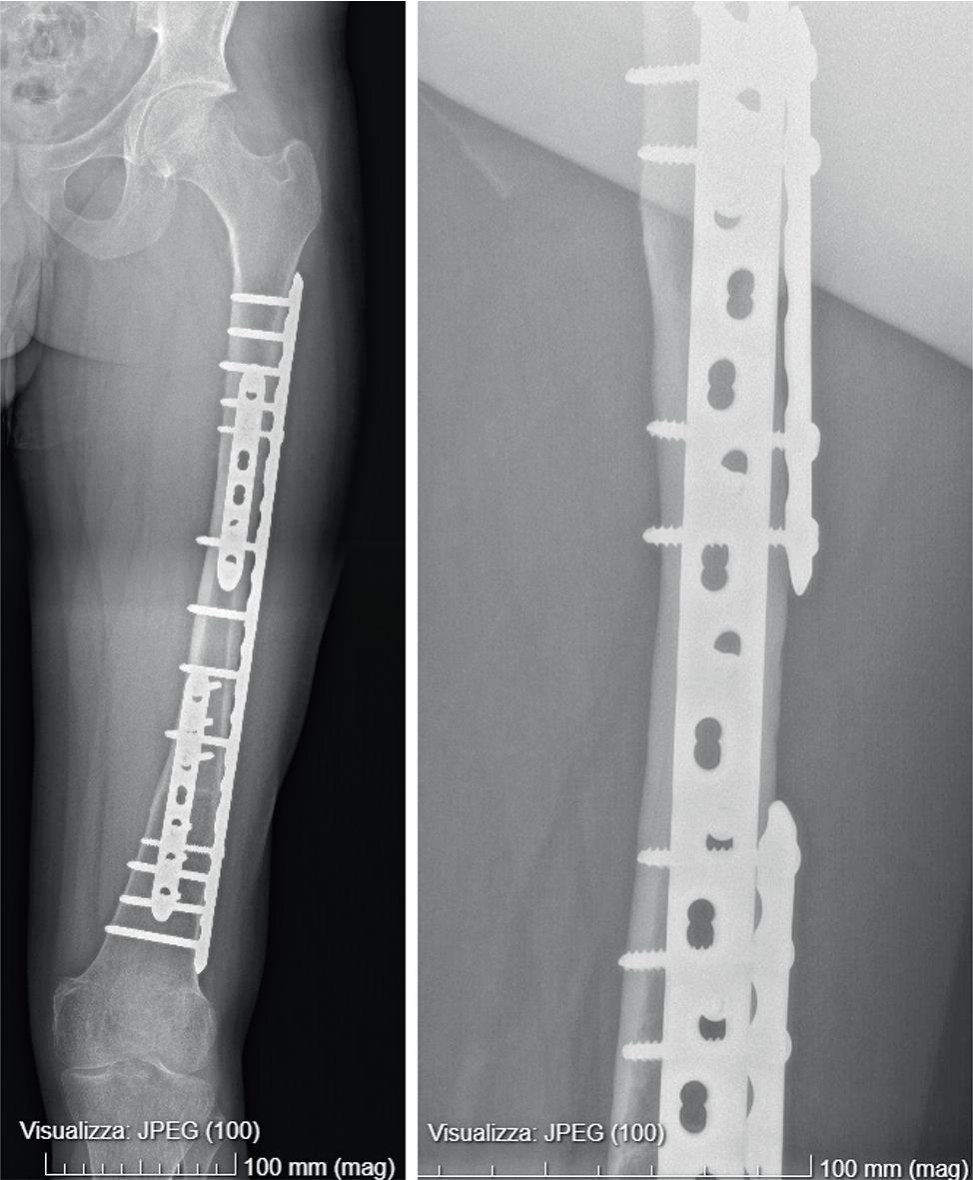
After a two‐year follow‐up x‐rays show femoral allograft union.

**Figure 8:**
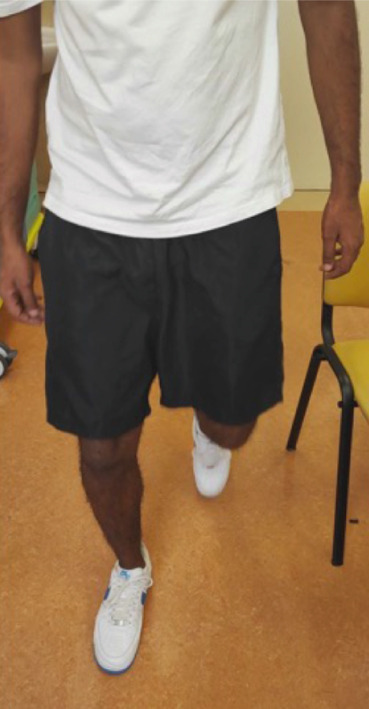
Patient′s image standing and walking without aids at the last follow‐up outpatient visit.

After a two‐year follow‐up, the patient is disease free and asymptomatic, walking with full weight‐bearing with radiographic evidence of allograft union (Figures 7 and 8).

## 3. Discussion

To date, less than 200 cases of PHME have been reported worldwide, predominantly in case reports [3]. As stated by Folpe in a recent review, there is no clear role for adjuvant chemotherapy or radiotherapy in the treatment of patients with PMHE, so it requires complete surgical excision for cure [13].

A literature review concerning PHME of bone was undertaken. Even if it was described in nearly 25% of reported cases, primary bone PHME is uncommon [14]. Dianat et al. shared a sacrum PHME treated by S1–S3 partial sacrectomy, tumor resection, argon beam laser diathermy, and allograft cancellous bone chips [9]. Kosemehmetoglu et al. reported five cases in the proximal femur, diaphysis of the tibia, distal radius and vertebra. All of them were treated with curettage, apart from the last one in which vertebrectomy was performed [10]. Otani et al. described a case with multiple bone lesions of the lower limb managed with curettage of one of them and systemic denosumab therapy [11]. Accordingly, Pasricha et al. presented a case with multicentric skeletal involvement successfully treated with denosumab [12].

Although PMHE exhibits some characteristic histological features, distinguishing it from other histologically similar vascular and epithelioid tumors—such as epithelioid hemangioendothelioma (EHE), epithelioid sarcoma (ES), and giant cell tumor of bone (GCT)—can be challenging. Accurate diagnosis is crucial, as these tumors differ significantly in their clinical behavior and malignant potential. A broad IHC panel incorporating several vascular and epithelial markers is recommended for differential diagnosis. However, overlapping immunoreactivity among these tumors can complicate the identification of PMHE. Among them, EHE represents the most important differential consideration because its histological features and clinical presentation closely resemble those of PMHE. Both typically demonstrate a fascicular proliferation of relatively bland spindle and/or epithelioid cells with eosinophilic cytoplasm.

Compared with EHE, PMHE demonstrates diffuse and strong nuclear FOSB positivity rather than CAMTA1 or TFE3 expression and typically exhibits a more solid and fascicular growth pattern with less pronounced vasoformation. Moreover, PMHE often presents as multiple musculoskeletal lesions involving skeletal bones, whereas EHE also tends to produce multifocal osseous lesions [14].

Unlike ES, PMHE lacks keratin AE1/AE3 and EMA coexpression with CD34, and it does not show loss of INI1. Moreover, in contrast to GCT of bone, PMHE is composed predominantly of plump spindle and epithelioid cells with scattered osteoclast‐like giant cells rather than a uniform distribution of mononuclear and multinucleated giant cells and lacks the typical GCT H3.G34 molecular alteration.

According to the literature, PMHE has a borderline biological behavior, with a high tendency to local recurrence but very low metastatic potential [3, 13–15]. Hence, a more conservative treatment can be adopted [10, 11]. Curettage is a valid option for PHME with limited cortical involvement. In our case, the presence of an undisplaced pathological fracture made intralesional curettage oncologically unsafe, therefore justifying a more aggressive approach. Diaphyseal site without evidence of joint involvement supported our final decision to perform an intercalary resection. A biological reconstruction with a massive allograft rather than a customized or modular intercalary prosthesis was preferred, in view of the patient′s age, to preserve bone stock and to avoid long‐term complications associated with prostheses. The option to augment the allograft with an autologous vascularized fibular graft was considered, but an intercalary allograft alone reconstruction was preferred by the patient. Our approach allowed us to find a compromise between surgical aggressiveness, disease eradication and functional result.

## 4. Conclusion

We report a case of PHME of bone treated with resection and intercalary allograft reconstruction. We strongly believe that sharing our experience will contribute to expanding our comprehension of this neoplasm, providing proper management and better outcomes for patients.

## Funding

No funding was received for this manuscript. Open access publishing facilitated by Università degli Studi di Firenze, as part of the Wiley ‐ CRUI‐CARE agreement.

## Consent

Written informed consent was obtained from the patient for publication of this case report and any accompanying images. All identifying information has been removed to protect patient privacy.

## Conflicts of Interest

The authors declare no conflicts of interest.

## Data Availability

The data that support the findings of this study are available on request from the corresponding author. The data are not publicly available due to privacy or ethical restrictions.

## References

[1] Jo V. Y. and Fletcher C. D. , WHO Classification of Soft Tissue Tumours: An Update Based on the 2013 (4th) edition, Pathology. (2014) 46, no. 2, 95–104, 10.1097/PAT.0000000000000050, 2-s2.0-84896748416, 24378391. 24378391

[2] Stacchiotti S. , Frezza A. M. , Blay J. Y. , Baldini E. H. , Bonvalot S. , Bovée J. V. M. G. , Callegaro D. , Casali P. G. , Chiang R. C. , Demetri G. D. , Demicco E. G. , Desai J. , Eriksson M. , Gelderblom H. , George S. , Gounder M. M. , Gronchi A. , Gupta A. , Haas R. L. , Hayes-Jardon A. , Hohenberger P. , Jones K. B. , Jones R. L. , Kasper B. , Kawai A. , Kirsch D. G. , Kleinerman E. S. , Le Cesne A. , Lim J. , Chirlaque López M. D. , Maestro R. , Marcos-Gragera R. , Martin Broto J. , Matsuda T. , Mir O. , Patel S. R. , Raut C. P. , Razak A. R. A. , Reed D. R. , Rutkowski P. , Sanfilippo R. G. , Sbaraglia M. , Schaefer I. M. , Strauss D. C. , Sundby Hall K. , Tap W. D. , Thomas D. M. , van der Graaf W. T. A. , van Houdt W. J. , Visser O. , von Mehren M. , Wagner A. J. , Wilky B. A. , Won Y. J. , Fletcher C. D. M. , Dei Tos A. P. , and Trama A. , Ultra-Rare Sarcomas: A Consensus Paper From the Connective Tissue Oncology Society Community of Experts on the Incidence Threshold and the List of Entities, Cancer. (2021) 127, no. 16, 2934–2942, 10.1002/cncr.33618. 33910263 PMC8319065

[3] Yang N. , Huang Y. , Yang P. , Yan W. , Zhang S. , Li N. , and Feng Z. , Clinicopathological Study of Pseudomyogenic Hemangioendothelioma, Diagnostic Pathology. (2023) 18, no. 1, 10.1186/s13000-023-01309-9, 36803395. PMC9940391 36803395

[4] Shackelford A. J. , Canterbury C. R. , Perrino M. A. , Wang J. , Philipone E. M. , and Peters S. M. , Oral Pseudomyogenic Hemangioendothelioma: Case Report and Review of the Literature, Head and Neck Pathology. (2020) 14, no. 4, 1134–1138, 10.1007/s12105-020-01137-z. 32016785 PMC7669923

[5] Tsubokawa N. , Harada H. , Taniyama D. , Uemura T. , Kuraoka K. , and Yamashita Y. , Epithelioid Sarcoma-Like Hemangioendothelioma on the Chest Wall, Asian Cardiovascular and Thoracic Annals. (2016) 24, no. 8, 814–817, 10.1177/0218492316664672, 2-s2.0-84991107001. 27493191

[6] Ge Y. , Lin X. , Zhang F. , Xu F. , Luo L. , Huang W. , Liu Z. , Liu Y. , and Li Z. , A Rare Case of Pseudomyogenic Hemangioendothelioma (PHE)/Epithelioid Sarcoma-Like Hemangioendothelioma (ES-H) of the Breast First Misdiagnosed as Metaplastic Carcinoma by FNAB and Review of the Literature, Diagnostic Pathology. (2019) 14, no. 1, 10.1186/s13000-019-0857-6, 2-s2.0-85069469043, 31311568. PMC6635997 31311568

[7] Thomas Z. , Georgy J. T. , Ponmar M. , Thumaty D. B. , Prabhu A. J. , and Singh A. , Metastatic Malignant Pseudomyogenic Hemangioendothelioma: An Exceedingly Rare Entity That Challenges Conventional Paradigms, International Journal of Surgical Pathology. (2025) 33, no. 4, 945–950, 10.1177/10668969241286068. 39350761

[8] Zhou J. , Yu D. H. , Chen X. R. , Wang J. Y. , and Cai S. Q. , Pseudomyogenic Hemangioendothelioma in the External Genitalia, Journal der Deutschen Dermatologischen Gesellschaft. (2024) 22, no. 5, 700–703, 10.1111/ddg.15353, 38581344. 38581344

[9] Dianat S. , Yousaf H. , Murugan P. , and Marette S. , Pseudomyogenic Hemangioendothelioma—A Case Report and Review of the Literature, Radiology Case Reports. (2019) 14, no. 10, 1228–1232, 10.1016/j.radcr.2019.06.029, 2-s2.0-85070584989, 31440320. 31440320 PMC6699196

[10] Kosemehmetoglu K. , Rekhi B. , Wakely P. E. , Pant V. , Dervisoglu S. , and Aydingoz U. , Pseudomyogenic (Epithelioid Sarcoma-Like) Hemangioendothelioma of Bone: Clinicopathologic Features of 5 Cases, Annals of Diagnostic Pathology. (2019) 41, 116–123, 10.1016/j.anndiagpath.2019.06.003, 2-s2.0-85067576689, 31233904. 31233904

[11] Otani S. , Nakayama R. , Sekita T. , Hirozane T. , Asano N. , Nishimoto K. , Sasaki A. , Okita H. , Morioka H. , Nakamura M. , and Matsumoto M. , Pseudomyogenic Hemangioendothelioma of Bone Treated With Denosumab: A Case Report, BMC Cancer. (2019) 19, no. 1, 10.1186/s12885-019-6072-8, 2-s2.0-85071766378, 31481040. PMC6724307 31481040

[12] Pasricha S. , Sharma A. , Pruthi M. , Durga G. , Jajodia A. , Gupta G. , Kamboj M. , Gupta M. , and Mehta A. , Multifocal Primary Pseudomyogenic Hemangioendothelioma of Bone Managed With Denosumab: A Rare Case With Diagnostic and Therapeutic Challenge, Journal of Cancer Research and Therapeutics. (2022) 18, no. 3, 817–819, 10.4103/jcrt.JCRT_1138_20, 35900565. 35900565

[13] Folpe A. L. , Vascular Tumors of Intermediate Malignancy: An update, Human Pathology. (2024) 147, 114–128, 10.1016/j.humpath.2024.01.014, 38360216. 38360216

[14] Sugita S. , Hirano H. , Kikuchi N. , Kubo T. , Asanuma H. , Aoyama T. , Emori M. , and Hasegawa T. , Diagnostic Utility of FOSB Immunohistochemistry in Pseudomyogenic Hemangioendothelioma and Its Histological Mimics, Diagnostic Pathology. (2016) 11, no. 1, 10.1186/s13000-016-0530-2, 2-s2.0-84981313762. PMC4982139 27515856

[15] Caballero G. A. and Roitman P. D. , Pseudomyogenic Hemangioendothelioma (Epithelioid Sarcoma-Like Hemangioendothelioma), Archives of Pathology & Laboratory Medicine. (2020) 144, no. 4, 529–533, 10.5858/arpa.2018-0395-RS, 31017450. 31017450

